# A prospective study of postmenopausal hormone use and ovarian cancer risk

**DOI:** 10.1038/sj.bjc.6603527

**Published:** 2006-12-19

**Authors:** K N Danforth, S S Tworoger, J L Hecht, B A Rosner, G A Colditz, S E Hankinson

**Affiliations:** 1 Channing Laboratory, Department of Medicine, Brigham and Women's Hospital and Harvard Medical School, Boston, MA, USA; 2 Department of Epidemiology, Harvard School of Public Health, Boston, MA, USA; 3 Department of Pathology, Beth-Israel Deaconess Medical Center, Boston, MA, USA; 4 Department of Biostatistics, Harvard School of Public Health, Boston, MA, USA

**Keywords:** hormone, ovarian cancer, postmenopausal, epidemiology

## Abstract

The relationship between postmenopausal hormone use (PMH) and ovarian cancer risk is unclear, particularly for specific hormone formulations, but recent studies suggest that there is a positive association. We conducted a prospective observational study with 82 905 postmenopausal women, including 389 ovarian cancers, in the Nurses' Health Study from 1976 to 2002. Compared with never users of PMH, both current and past users of ⩾5 years had a significantly elevated risk of ovarian cancer (RR=1.41, 95% confidence interval (CI) 1.07–1.86 and relative risk (RR)=1.52, 95% CI 1.01–2.27, respectively). Examined by hormone type in continuous years, use of unopposed estrogen was associated with a significant increase in the risk of epithelial ovarian cancer (*P* for trend <0.001; RR for 5-year increment of use=1.25, 95% CI 1.12–1.38). Use of estrogen plus progestin (RR for 5-year increment of use=1.04, 95% CI 0.82–1.32) was not significantly associated with ovarian cancer risk. Generally, results were similar for serous tumours (RR for 5-year increment of unopposed estrogen use=1.23, 95% CI 1.07–1.40) and slightly stronger for endometrioid tumours (RR for 5-year increment of unopposed estrogen use=1.53, 95% CI 1.20–1.94). Recency of use was not significantly associated with ovarian cancer risk, but statistical power was limited here.

Ovarian cancer is the fifth most common cause of cancer mortality among women in the United States (American Cancer Society, 2006), and yet few truly modifiable factors have been established. Postmenopausal hormone (PMH) use has been examined as a potential risk factor for ovarian cancer in several studies, but reviews (IARC Working Group on the Evaluation of Carcinogenic Risks to Humans *et al*, 1999; Riman *et al*, 2004; Farquhar *et al*, 2005) and meta-analyses (Garg *et al*, 1998; Coughlin *et al*, 2000) have found studies to be inconsistent, as did the US Preventive Services Task Force review of higher-quality studies (USPSTF, 2005). Recently, unopposed estrogen use has been positively associated with ovarian cancer risk or mortality in several cohort studies (Rodriguez *et al*, 2001; Lacey *et al*, 2002, 2006; Folsom *et al*, 2004). Although estrogen plus progestin use also has been examined in multiple studies (Lacey *et al*, 2002, 2006; Riman *et al*, 2002; Sit *et al*, 2002), only one cohort study reported a substantial number of cases among long-duration estrogen plus progestin users, finding a significant increased risk of ovarian cancer (Lacey *et al*, 2006).

We evaluated the association between PMH use and ovarian cancer in the prospective Nurses' Health Study (NHS) over 26 years of follow-up; we examined duration, recency of use, and PMH type for all ovarian tumours, as well as by histologic tumour type.

## MATERIALS AND METHODS

### Study cohort

The NHS began in 1976, when 121 701 female registered nurses in 11 US states completed a self-administered, mailed questionnaire. At enrolment, participants were 30–55 years old. Subsequently, follow-up questionnaires were mailed biennially to obtain updated exposure and disease information. Information on deaths was obtained through the post office, relatives and linkages with the National Death Index. Through 2002, these methods yielded a follow-up rate of 93.7% of potential person-years. The study was approved by the Institutional Review Board of Brigham and Women's Hospital.

### Study population

The study population was restricted to postmenopausal women. A validation study in the NHS found self-reported menopause to have high reproducibility (Colditz *et al*, 1987). Women whose menopausal status was missing, or who reported a hysterectomy without bilateral oophorectomy, contributed person-time from the age at which natural menopause occurred for 90% of the cohort (54 years for current smokers, 56 years for past or never smokers).

In 1976, there were 24 443 postmenopausal women in the NHS. Participants were excluded if they reported radiation as the reason for menopause (*n*=209), a bilateral oophorectomy (*n*=8506), or a diagnosis of cancer other than non-melanoma skin cancer (*n*=514) before the start of follow-up. Women missing exposure or covariate information (*n*=1074) were excluded (details below), leaving 14 140 eligible women in the baseline population.

Women subsequently entered the study population as they became postmenopausal, provided that radiation or bilateral oophorectomy was not the cause of menopause. Participants were censored at the earliest of: (1) development of ovarian cancer, (2) report of any cancer other than non-melanoma skin cancer, (3) death, or (4) the end of the study period, 6/1/2002. Person-time with missing exposure or covariate information also was excluded. From 1976 to 2002, 82 905 postmenopausal women accumulated a total of 966 017 person-years.

### Postmenopausal hormone use

PMH use was assessed in every questionnaire. In 1976, users reported their total duration of use. As 72% of users of a known type reported use of unopposed estrogen in 1978 (when such details were first collected), PMH use in 1976 was classified as unopposed estrogen. This classification probably resulted in a small amount of misclassification of other types of PMH; however, in a sensitivity analysis, we also re-coded PMH use in 1976 to other/unknown type of hormone.

### Ovarian cancer

Incident cases of ovarian cancer were identified through responses to biennial questionnaires or death certificates and confirmed by medical record review. From 1976 to 2002, there were 760 reported cases of postmenopausal ovarian cancer. We were unable to obtain medical records for 71 (9.3%) and did not confirm the diagnosis upon medical record review for 135 (17.8%) women. Of the 554 confirmed cases, 492 were epithelial tumours (88.8%). After applying exclusion criteria (e.g., prior diagnosis of another cancer), we were left with 389 cases of primary epithelial ovarian cancer.

Histologic type, as coded from pathology reports by a gynecologic pathologist (JLH), had the following distribution: 233 serous/poorly differentiated (hereafter referred to as serous), 60 endometrioid, 35 mucinous, 19 clear-cell and 42 other/unknown subtype. Of the 389 cases, 353 were invasive and 36 were of low malignant potential (18 serous, 15 mucinous, 2 endometrioid and 1 clear cell).

### Covariates

Age and time period were used as stratification variables in the Cox proportional hazards models. Based on previous literature, the following covariates were forced into the multivariate models: duration of oral contraceptive use (continuous), parity (continuous), tubal ligation (yes/no), age at natural menopause (continuous) and age at menarche (<12, 12, 13, 14, ⩾15 years). The complete case method (restricting the analysis to participants with data on the exposure and all covariates) was used, except that women with a hysterectomy were included; the population was restricted to women with natural menopause in secondary analyses.

In addition, the following potential confounders were not included in the final models: vigorous physical activity, smoking, alcohol consumption, caffeine intake, lactose/galactose consumption, perineal talc use, breastfeeding, simple hysterectomy, use of non-steroidal anti-inflammatory medications other than aspirin and family history of breast cancer. Data on family history of ovarian cancer, first collected in 1992, was evaluated as a potential confounder by examining its distribution across PMH categories in 1992. Fat intake and body mass index (BMI) were not considered confounders because they were not associated with postmenopausal ovarian cancer in previous NHS analyses (Bertone *et al*, 2002; Fairfield *et al*, 2002). However, BMI and having an intact reproductive system (no tubal ligation or hysterectomy) were evaluated as potential effect modifiers.

### Data analysis

Multivariate Cox proportional hazards regression models were used to estimate RRs and 95% CIs. The association with ovarian cancer was examined for status of PMH use (never, past and current), total duration and time since last use. In addition, analyses were performed by type-specific duration in continuous years, simultaneously including all PMH types in the models (unopposed estrogen, estrogen plus progestin, other PMH). Results have not been presented for the ‘other PMH’ group because it represents a heterogeneous group of hormones, including non-conjugated estrogens, patch hormones, and vaginal hormones, as well as person-time for which hormone type was not reported.

Primary analyses included all tumours (invasive and low malignant potential), but sensitivity analyses restricted cases to invasive tumours. Separate analyses were performed for serous and endometrioid tumour subtypes, but owing to small numbers, those for mucinous tumors (*n*=35) were adjusted only for age, and clear cell tumors (*n*=19) were not evaluated.

## RESULTS

### Description of the study population

Population characteristics are presented for 1992 (Table 1), the approximate midpoint of the study, and the first year in which family history of ovarian cancer was collected. The mean age among study participants in 1992 was 61.2 years. The average duration of hormone use was longer among current unopposed estrogen users than current estrogen plus progestin users (9 vs 6 years, respectively). Compared to never users, PMH users were more likely to have used oral contraceptives or had a simple hysterectomy. As expected, current users of unopposed estrogen were substantially more likely to have had a hysterectomy than current users of estrogen plus progestin. Correspondingly, tubal ligation was more common among users of estrogen plus progestin than users of unopposed estrogen. The distribution of other risk factors was generally similar across PMH classifications. There was no substantial variation by family history of ovarian cancer across exposure groups.

### Multivariate results

Results from the age-adjusted and multivariate models were nearly identical, indicating minimal confounding by other risk factors (Table 2). Neither current (RR=1.24, 95% CI 0.97–1.59) nor past (RR=1.00, 95% CI 0.77–1.31) use of PMH was significantly associated with ovarian cancer risk compared with never use. For serous tumours, current use of PMH was associated with a significant increase in risk (RR=1.43, 95% CI 1.04–1.96) compared to never use. Risk was non-significantly increased for endometrioid tumors for current (RR=1.61, 95% CI 0.85–3.05) and past (RR=1.68, 95% CI 0.85–3.33) use compared with never use. Results were similar when models were restricted to invasive tumours (data not shown).

When analyses were stratified by total duration of PMH use (<5 and ⩾5 years), increased risk was observed among both current (RR=1.41, 95% CI 1.07–1.86) and past (RR=1.52, 95% CI 1.01–2.27) users of five or more years compared with never users (Table 3). Results were similar for serous tumors, although statistically significant only for current users of greater than five years (RR=1.66, 95% CI 1.17–2.36). Results generally were stronger for endometrioid tumours, although the increased risk was statistically significant for past (RR=3.59, 95% CI 1.41–9.14), but not current (RR=1.86, 95% CI 0.89–3.91), users of 5 or more years compared with never users.

Combining current and past use, continuous years of unopposed estrogen use were significantly associated with increased risk (*P* for trend <0.001, RR=1.25 for a 5-year increment of use, 95% CI 1.12–1.38), whereas continuous years of estrogen plus progestin use were not (*P* for trend=0.77, RR=1.04 for a 5-year increment of use, 95% CI 0.82–1.32) (Table 4). Results were similar when hormone use in 1976 was re-classified as unknown/other type (data not shown) and when terms were added for past and current use (data not shown). The increased risk observed with continuous years of unopposed estrogen use was generally similar for serous tumours (RR for 5-year increment of unopposed estrogen use=1.23, 95% CI 1.07–1.40) and slightly stronger for endometrioid tumours (RR for 5-year increment of unopposed estrogen use=1.53, 95% CI 1.20–1.94), although there was a limited number of cases for the endometrioid analysis. When mucinous tumours were examined in age-adjusted analyses, point estimates suggested that past (RR=0.72, 95% CI 0.30–1.76) and current (RR=0.72, 95% CI 0.31–1.67) users had a reduced risk compared with never users, although results were not significant and based on few cases.

In analyses restricted to women exclusively using one hormone formulation, unopposed estrogen use of five or more years was associated with an increased risk compared with never use (RR=2.04, 95% CI 1.41–2.97), whereas estrogen plus progestin use of five or more years was not (RR=0.93, 95% CI 0.47–1.83) (Table 5). However, there were only ten cases among estrogen plus progestin users of ⩾5 years. Results were similar for unopposed estrogen use among women reporting a hysterectomy and estrogen plus progestin use among women with intact uteri (data not shown).

Time since last use was not significantly associated with risk. Neither users who quit within the previous three years (RR=1.04, 95% CI 0.71–1.53) nor those who stopped over three years ago (RR=0.86, 95% CI 0.60–1.22) were at a significantly increased risk of ovarian cancer. Based on the significant association observed with duration, a recency effect would most likely be seen primarily among long-term users. Although limited by small numbers, when we examined the effects of recency and duration together, point estimates suggested an increase in risk among users of five or more years that decreased over time (RR=1.62 among the most recent quitters and RR=1.35 among those who quit over 3 years ago, with 17 and 11 cases respectively).

No substantial differences were observed when stratifying on BMI (<25, 25–29, ⩾30 kg m^−2^) or an intact reproductive system (i.e., no prior hysterectomy or tubal ligation) (data not shown). Results were also similar among women reporting natural menopause, although the association with unopposed estrogen use was slightly stronger (RR=1.40 for a 5-year increment of use, 95% CI 1.09–1.80).

## DISCUSSION

In this prospective study, we observed a positive association between long duration of PMH use and ovarian cancer risk, regardless of whether use was current or past. More specifically, duration of unopposed estrogen use was positively associated with risk, whereas estrogen plus progestin use was not; the association was stronger for endometrioid tumours, although numbers were small.

In a collaborative re-analysis of 12 case–control studies, no association was found with duration of PMH use in either hospital-based (OR=0.90 for a 5-year increment of use, *P*=0.37) or population-based (OR=1.10 for a 5-year increment of use, *P* = 0.21) studies (Whittemore *et al*, 1992). Meta-analyses (Garg *et al*, 1998; Coughlin *et al*, 2000) and certain case–control studies (Weiss *et al*, 1982; Riman *et al*, 2002) also failed to find a significant trend with duration of use, although some found a positive association (Risch, 1996) or suggested a positive trend (Kaufman *et al*, 1989; Bosetti *et al*, 2001).

Recently, four prospective studies found that longer durations of PMH use were associated with ovarian cancer risk or death (Rodriguez *et al*, 2001; Lacey *et al*, 2002; Folsom *et al*, 2004; Lacey *et al*, 2006). In two, ovarian cancer risk (Lacey *et al*, 2006) and mortality (Rodriguez *et al*, 2001) were increased among unopposed estrogen users of ⩾10 years but not among users of <10 years; elevations in mortality were similar for current and past long-duration users (Rodriguez *et al*, 2001). Two other cohort studies observed increases in risk with shorter durations of use: in the Breast Cancer Detection Demonstration Project (BCDDP, *n*=329 cases), unopposed estrogen use was significantly associated with risk (RR=1.40 for 5 years use, converted from a 1-year estimate), with similar results for recent and former long-duration users (Lacey *et al*, 2002). In another prospective study, current users of unopposed estrogens for >5 years had a significantly elevated risk (RR=2.53, 95% CI 1.44–4.45, *n*=16 cases); although risk was not increased among long-duration past users, there were only four cases (Folsom *et al*, 2004). Findings from recent case–control studies also generally support a positive association with long-duration PMH use (Glud *et al*, 2004; Mills *et al*, 2004; Pike *et al*, 2004; Riman *et al*, 2004; Moorman *et al*, 2005).

We found a strong association with duration of PMH use and risk among current and past users of five or more years duration. With both duration and status of use (never, past, current) in the same model, only duration was statistically significant (data not shown). Overall, the significant increase in risk appeared to be driven largely by duration rather than by status of use. This contrasts with breast cancer PMH findings, where the increased risk is confined to current users (Colditz *et al*, 1995; Collaborative Group on Hormonal Factors in Breast Cancer, 1997; Beral *et al.*, 2003), although the comparison is limited by the relative paucity of data on recency effects for ovarian cancer.

Only recently have studies had sufficient case numbers to evaluate associations for estrogen and progestin use. A Swedish case–control study (*n*=655 cases) found that use of estrogen plus sequential progestin was associated with an increased risk, whereas estrogen plus continuous progestin was not (Riman *et al*, 2002). However, results from the women's health initiative (WHI), a randomized clinical trial of estrogen plus continuous progestin (*n*=32 cases), were consistent with an increase in risk (RR=1.58, 95% CI 0.77–3.24), although not statistically significant (Anderson *et al*, 2003). Another cohort study found an increased risk associated with both sequential (RR=3.09, 95% CI 1.68–5.68; *n*=13 cases) and continuous (RR=1.82, 95% CI 1.03–3.23; *n*=15 cases) estrogen plus progestin use of ⩾5 years in women without a hysterectomy (Lacey *et al*, 2006).

In contrast, a cohort study in Norway found no association with use of estrogen plus progestin (RR=1.5, 95% CI 0.9–2.6; *n* = 23 cases) (Bakken *et al*, 2004), and nor did the BCDDP study. Data suggested that unopposed estrogen, followed by estrogen plus progestin, was associated with risk; however, the effects of the different hormones could not be disentangled (Lacey *et al.*, 2002). In our study, when simultaneously including terms for years of unopposed estrogen, estrogen plus progestin and other PMH use, only unopposed estrogen use was significantly associated with risk; results were consistent among users of a single hormone type. However, duration of estrogen plus progestin use was on an average shorter than use of unopposed estrogen, and the upper confidence limits were similar to those observed for unopposed estrogen use. Further studies about long-duration estrogen plus progestin use are therefore needed, given its more recent introduction to the market, particularly those focused on sequential or continuous hormone regimens.

Among the few analyses by histologic type, one suggested that PMH use might specifically increase risk of endometrioid tumours (Weiss *et al*, 1982). Other prospective studies either have not examined tumour subtype (Rodriguez *et al*, 2001; Anderson *et al*, 2003; Bakken *et al*, 2004; Folsom *et al*, 2004; Lacey *et al*, 2006) or had incomplete information on histology (Lacey *et al*, 2002). Despite limited power, in our study, the association with unopposed estrogen use appeared slightly stronger for endometrioid tumours. In age-adjusted analyses, point estimates suggested PMH use might decrease the risk of mucinous tumours, consistent with some (Weiss *et al*, 1982; Risch, 1996), but not all (Riman *et al*, 2002), previous reports. Although statistical comparisons between the subtypes were precluded by sample size, our findings are consistent with epidemiologic and biologic data. Endometrioid tumours are histologically similar to endometrial tissue (Kumar *et al*, 1997), and unopposed estrogen use increases the risk of endometrial cancer (Fraser *et al*, 1998). Mucinous tumors are sub-classified as those that resemble colonic or endocervical epithelium (Kumar *et al*, 1997). PMH use has been associated with decreased colon cancer risk (Nelson *et al*, 2002) but not with altered cervical cancer risk (Weiss and Hill, 1996).

The mechanism by which PMH might affect ovarian cancer risk is unknown. One theory posits that high levels of gonadotropins increase risk, implying that PMH use might decrease risk by reducing these levels (follicle-stimulating hormone (FSH) and leutinising hormone (LH)), but as the declines associated with PMH use are small, the benefits might be outweighed by estrogen-induced proliferation of ovarian cells (Cramer and Welch, 1983; Fraser *et al*, 1998); it has been estimated that as many as 60% of ovarian tumours are estrogen receptor-positive (Cunat *et al*, 2004). Breast cancer research also suggests that estrogen may be directly genotoxic (Ho, 2003). Although it is premature to conclude that estrogen plus progestin use is unassociated with ovarian cancer risk, particularly given the conflicting findings, research on ovulating macaques suggests that progesterone offsets the effect of unopposed estrogen use by increasing apoptosis in the ovary (Rodriguez *et al*, 1998), possibly by altering levels of TGF-*β*, a regulator of apoptosis (Rodriguez *et al*, 2002). The progesterone in these animal studies may have different effects from the progestins commonly used in PMH formulations, but a mechanism is suggested, given that progesterone receptors are normally found in ovarian epithelium (Risch, 1998).

Our analysis has several strengths. The NHS is one of only a few prospective studies of PMH use and ovarian cancer, and associations could be examined by hormone type. Information on exposures and confounders is updated through biennial questions, and follow-up of the cohort is high. The nurses are a relatively homogenous group, with similar education and access to health care, reducing concerns about confounding. Although family history of ovarian cancer was first collected in 1992, this did not vary substantially across exposure. Histologic tumour type was coded by a gynecologic pathologist and was available for most cases.

Nurses' Health Study (NHS) participants are not a representative sample of the general population. While it is unlikely that the observed associations would differ in other women, studies covering different race/ethnicity and socioeconomic status are warranted. Generalisability may also be limited by the variations in PMH formulations across countries. We had limited power to look at non-oral formulations of PMH, which are more commonly used outside the US (Ho, 2003). Small numbers prevented evaluation of different regimens of estrogen plus progestin and limited the analysis of recency of use.

In conclusion, we found that use of PMH was positively associated with risk of epithelial ovarian cancer. With other recent studies, our findings suggest that women should be counseled about the potential long-term increase in ovarian cancer risk with extended use of unopposed estrogen. Evidence is insufficient to say whether estrogen plus progestin or very short durations of unopposed estrogen use are associated with risk. Available findings indicate that ovarian cancer is one of several conditions that should be considered by women when weighing the risk and benefits of PMH use.

## Figures and Tables

**Table 1:** Age and age-standardised characteristics by postmenopausal hormone use and hormone type in the Nurses' Health Study, 1992^a^

	**PMH status**			**Current PMH users**	
	**Never user (*n*=20 853)**	**Past user (*n*=10 053)**	**Current user (*n*=16831)**	**Estrogen only (*n*=4315)**	**Estrogen+Progestin (*n*=7394)**
Age, mean, years	61	64	60	62	58
Duration of PMH use, mean, years	0	3	7	9	6
					
*Duration of OC use* (%)					
Never	66	58	55	54	52
< 3 years	18	23	22	23	22
⩾3 years	17	20	23	23	26
					
*Parity*					
Parous women (%)	94	93	93	94	93
Mean no. of children (among parous)	3	3	3	3	3
Family^b^ history of ovarian cancer (%)	3	3	2	3	3
Had a simple hysterectomy (%)	5	12	19	47	2
Had a tubal ligation (%)	13	14	14	10	17
Age at menarche, mean, years	13	13	13	13	13
Age at natural menopause, mean, years	50	49	50	49	50

Abbreviations: PMH, postmenopausal hormone; OC, oral contraceptive.

a Characteristics are presented for the 47 737 nurses who met the study eligibility criteria in 1992; all factors except age were age-standardised in 5-year intervals.

b Mother or sister had ovarian cancer according to nurse's response to questionnaire.

**Table 2:** Use of postmenopausal hormones and epithelial ovarian cancer risk; all cases combined and by histologic type^a^

	**Never user**	**Past user**	**Current user**
*All epithelial ovarian tumours*			
No. of cases	167	88	134
Person-years	455 200	210 778	300 039
Age-adjusted RR (95% CI)	1.00 (referent)	1.00 (0.77, 1.31)	1.23 (0.97, 1.57)
Multivariate^b^ RR (95% CI)	1.00 (referent)	1.00 (0.77, 1.31)	1.24 (0.97, 1.59)
			
*Serous tumours*			
No. of cases	96	51	86
Multivariate^b^ RR (95% CI)	1.00 (referent)	0.98 (0.69, 1.40)	1.43 (1.04, 1.96)
			
*Endometrioid tumours*			
No. of cases	21	16	23
Multivariate^b^ RR (95% CI)	1.00 (referent)	1.68 (0.85, 3.33)	1.61 (0.85, 3.05)

Abbreviations: RR, relative risk; CI, confidence interval.

a Results by histologic type are presented for serous/poorly differentiated and endometrioid tumors only due to small numbers for the other histologic types.

b Adjusted for: age, parity, duration of oral contraceptive use, tubal ligation, age at natural menopause, age at menarche.

**Table 3:** Total duration of postmenopausal hormone use and risk of epithelial ovarian cancer; all cases combined and by histologic type^a^

		**Past user**		**Current user**	
	**Never user**	**<5 years use**	**⩾5 years use**	**<5 years use**	**⩾5 years use**
*All epithelial ovarian tumours*					
No. of cases	167	57	31	40	94
Person-years	455 200	164 558	46 220	129 669	170 370
Age-adjusted RR (95% CI)	1.00 (referent)	0.87 (0.64, 1.18)	1.50 (1.01, 2.22)	1.00 (0.70, 1.43)	1.38 (1.06, 1.82)
Multivariate^b^ RR (95% CI)	1.00 (referent)	0.88 (0.64, 1.19)	1.52 (1.01, 2.27)	1.01 (0.70, 1.44)	1.41 (1.07, 1.86)
					
*Serous tumours*					
No. of cases	96	32	19	23	63
Multivariate^b^ RR (95% CI)	1.00 (referent)	0.83 (0.55, 1.25)	1.60 (0.95, 2.68)	1.09 (0.68, 1.75)	1.66 (1.17, 2.36)
					
*Endometrioid tumours*					
No. of cases	21	9	7	8	15
Multivariate^b^ RR (95% CI)	1.00 (referent)	1.25 (0.56, 2.80)	3.59 (1.41, 9.14)	1.38 (0.59, 3.25)	1.86 (0.89, 3.91)

Abbreviations: RR, relative risk; CI, confidence interval.

a Results by histologic type are presented for serous/poorly differentiated and endometrioid tumours only due to small numbers for the other histologic types.

b Adjusted for age, parity, duration of oral contraceptive use, tubal ligation, age at natural menopause and age at menarche.

**Table 4:** Continuous years of estrogen and estrogen plus progestin use and risk of epithelial ovarian cancer; all cases combined and by histologic type^a^

	**Continuous RR converted to 5-year increment**	
	**Estrogen only**	**Estrogen plus progestin**
*All ovarian tumours*		
Cases^b^	137	82
Age-adjusted RR (95% CI)	1.23 (1.11, 1.35)	1.05 (0.82, 1.33)
Multivariate^c^ RR (95% CI)	1.25 (1.12, 1.38)	1.04 (0.82, 1.32)
		
*Serous tumours*		
Cases^b^	89	49
Multivariate^c^ RR (95% CI)	1.23 (1.07, 1.40)	1.12 (0.84, 1.51)
		
*Endometrioid tumours*		
Cases^b^	23	15
Multivariate^c^ RR (95% CI)	1.53 (1.20, 1.94)	1.04 (0.53, 2.03)

Abbreviations: RR, relative risk; CI, confidence interval; PMH, postmenopausal hormone.

a Results by histologic type are presented for serous/poorly differentiated and endometrioid tumours only due to small numbers for the other histologic types.

b Some participants (and therefore cases) contribute to multiple categories simultaneously because they used estrogen only, estrogen plus progestin and/or other PMH formulations.

c Adjusted for age, parity, duration of oral contraceptive use, tubal ligation, age at natural menopause and age at menarche.

**Table 5:** Risk of epithelial ovarian cancer among exclusive users of one hormone type (estrogen only and estrogen plus progestin)

	**Estrogen only^a^**		**Estrogen plus progestin^a^**	
	**<5 years use**	**⩾5 years use**	**<5 years use**	**⩾5 years use**
*All epithelial ovarian tumours*				
No. of cases	39	43	22	10
Age-adjusted (95% CI)	0.94 (0.66, 1.34)	1.81 (1.28, 2.56)	1.03 (0.64, 1.67)	0.92 (0.47, 1.79)
Multivariate^b^ RR (95% CI)	0.98 (0.68, 1.40)	2.04 (1.41, 2.97)	1.03 (0.64, 1.66)	0.93 (0.47, 1.83)

Abbreviations: RR, relative risk; CI, confidence interval; PMH, postmenopausal hormone.

a Models include never PMH users and users of one hormone type only (exclusive users of unopposed estrogen and exclusive users of estrogen plus progestin).

b Adjusted for age, parity, duration of oral contraceptive use, tubal ligation, age at natural menopause and age at menarche.
